# German System of National Insurance

**Published:** 1889-10-05

**Authors:** 


### The German System of National Insurance.

PROVISION FOR TWELVE MILLION PERSONS.

The system of national insurance initiated in Germany, says the *Pall Mall Gazette*, is the greatest scheme of the kind ever devised by a State, and is significant of the Socialist tendencies of the times. The law aims at providing every labourer, workman, or servant in the empire with some material assistance when rendered unable to earn his living, owing to mental or bodily infirmity, or as soon as he has reached the age of 70, and it is calculated that about 12,000,000 persons will come within the scope of the Act. The State has undertaken to contribute a large portion of the sums required to meet the allowances, which, it is estimated, will eventually amount to £12,520,000 per annum, with £600,000 per annum in addition as the costs of administration. Objections were taken to this State subsidy while the Bill was under discussion, because, on the one hand, it introduced a dangerous Socialist principle, and that it would deprive the measure of much of its virtue by giving it a pauperising tendency; and, on the other hand, because the sums which would have to be raised to meet it would in all probability lead to additional indirect taxation, the burden of which, falling principally upon the poorer classes, would render the State subsidy a bane to them rather than a blessing. The Government, however, considered that the money could never be raised by direct contributions, more especially as the scheme is taken out of the ordinary commercial category by the decision to grant allowances for old age immediately on the Act coming into force, and those for infirmity at the expiration of one year from that date. As it was also imperative to form a sinking fund, a State subsidy became *ipso facto* a necessity. The principle was therefore admitted, and the amount of the State contribution was fixed at £2 10s. yearly for every allowance. It is reckoned that the State subsidy will amount to £320,000 the first year, and gradually rise till it reaches £3,450,000 in the eightieth year of insurance, when it is supposed that an approximate balance will for the first time be established between the number of allowances to be paid and the amount of capital collected to meet insurance liabilities, and the State subsidy being no longer necessary will be gradually extinguished.

The Persons Embraced.

A translation of the Act made by Mr. Esme Howard and forwarded by Sir Edward B. Malet to Lord Salisbury has been published, and from this paper and an able summary accompanying it we learn that it is obligatory upon all such persons to be insured upon completing the sixteenth year of their life as:

1. Are employed as workmen, assistants, apprentices, or servants, and receive for their service a payment or wage.

2. Are engaged in business as assistants in shops and apprentices (excepting assistants and apprentices of apothecaries), who are in receipt of payment or wage, but whose regular yearly earnings do not exceed £100. And

3. Persons employed for payment or wage as members of the crew, etc., of German ships, whether for sea or for inland navigation.

The provisions may also be extended to persons who do not employ regularly at least one paid workman, or small masters who are employed by others. Officials in the service of the State and persons serving as soldiers who are also employed as servants, are not liable to compulsory insurance. The insured have been divided into four wage classes, as follows:

| | | |
|---|---|---|
| Class 1: | Yearly wages up to | £17 10s. |
| ,, 2 | ,, ,, | 27 10s. |
| ,, 3 | ,, ,, | 42 10s. |
| ,, 4 | ,, over | 42 10s. |

The Contributions.

The contributions payable by employer and insured together in equal shares are fixed for the first ten years after the law comes into force at:

| | | | |
|---|---|---|---|
| Class 1 | 14 pf. | weekly | (or 1¼d.) |
| ,, 2 | 20 ,, | ,, | (or 2¼d.) |
| ,, 3 | 24 ,, | ,, | (or 3d.) |
| ,, 4 | 30 ,, | ,, | (or 3¼d.) |

These rates of contribution are calculated to be sufficient to meet all necessary expenditure during the first ten years, even under the most adverse conditions. After that period they may be settled anew every five years, according to circumstances. And it is reckoned that the highest rate of contributions, after eighty years' time, when the equilibrium has been reached, and the State subsidy has been discontinued, will not exceed 2½d. per week for the first class and 8d. for the fourth (half being paid by the employer and half by the insured).

Allowances for Infirmity and Age.

Every person is entitled to an allowance for infirmity—without taking his age into account—who has become confirmedly unfit for work. A man may be considered as unfit when, in consequence of his bodily or mental condition, he is not in a position to earn by means of labour, paid in a manner corresponding to his powers and capabilities, a sum which amounts to at least one-sixth of the average standard of wages, according to which contributions have been paid for him during the previous five contributory years. Every insured person is entitled to an allowance for old age, without necessarily proving that he is incapable of work, who has completed the seventieth year of his life. The insured who, without being confirmed invalids, are unfit for work during an entire year are also entitled to receive an allowance for infirmity during the further continuation of their inability to work. Such insured persons as have brought upon themselves either purposely or by committing any criminal action—declared as such by a court of law—a condition of unfitness to work, have no claim to an allowance for infirmity.

In order to become entitled to an infirm allowance, it is necessary to have paid contributions during five contributory years, except as regards cases of infirmity setting in within the first five years, but after the first year subsequent to the Act coming into force, for which cases special provision is made. Forty-seven weekly contributions are reckoned as completing the contributory year, this diminution on the calendar year being made in favour of persons who cannot be in regular employment throughout the year. The infirm allowances range, after five contributory years, from £5 14s. 9d. per annum in the lowest wage class to £7 0s. 2½d. in the highest, and after fifty contributory years, from £7 17s. in the lowest class to £20 15s. 6d. in the highest.

A period of thirty contributory years, equal to 1,410 contributory weeks, is prescribed for the attainment of an age allowance, the payments in the several classes being as follows:

| | | | |
|---|---|---|---|
| Class 1 | £5 6 5 | Class 3 | £8 2 10 |
| ,, 2 | 6 14 7 | ,, 4 | 9 11 0 |

These annual payments are very small, and it was suggested that the contributions should be raised in order to make the annuities somewhat more substantial; but the Government decided that it would be better to proceed cautiously at first, so as not to impose too heavy a burden on the labouring classes at the start, and it is always open to raise the amount at some future period. Besides, it is pointed out that the lowest allowance for old age, £5 6s. 5d.—"which to our English ears sounds a very meagre pittance—is equivalent to more than a third of the yearly average earnings of that wage class for which such allowance is granted; so that, taking into consideration the conditions of life indicated by this very low rate of wages, the allowance, however humble, is by no means to be despised. The Act, moreover, never aimed at providing the aged and incapacitated with sufficient means to enable them to live independently; these allowances should rather be considered as an addition to whatever means of subsistence the pensioner may otherwise possess; while, even if he has no other resources of his own, whereas formerly he must have been a burden on his family or the parish, his regular allowance will now be generally regarded as an agreeable increment of the household income, and will make him a welcome guest at the family hearth." In the case of an insured person dying before coming into the receipt of an allowance, that part which was deducted from his wages may be restored to his widow and orphans. A woman on marrying may also claim the restitution of her payments.

Collection and Administration.

The difficulty of collecting the minute contributions of twelve million persons weekly has been very simply solved. Four kinds of stamps, corresponding in value to the different contributions of the four wage classes, will be issued. The employer, who must purchase these stamps beforehand, will on each pay-day deduct one-half of the contributions for the previous week from the wages of the insured in his employ (the other half being payable by himself) and then paste an insurance stamp, corresponding in value to the full contribution, on to the receipt card of the insured. These receipt cards contain forty-seven spaces, the number of weeks of the contributory year. The cards, duly numbered and dated, are collected and stored away, so that each person's contributions can be easily traced back. The payment of allowances is effected in an equally simple way through the instrumentality of the postal authorities.

Insurance institutes will be established throughout the empire for the administration and execution of the provisions of the Act. All has been done to reduce the expenses to a minimum, and it has been of immense advantage in this respect that the services both of the postal administration and of the lower administrative authorities have been secured to assist in carrying out the provisions of the Act. The costs of administration are calculated at 1s. per head, or £600,000 per annum.

Existing Insurance Funds.

All already existing benefit and insurance funds, whether simply local or for trades or Government services, are left untouched by this law, which officially recognises their existence, and relieves their members of compulsory insurance provided that the regulations of such funds meet certain conditions, while the Act works conjointly with these funds as regards persons insured at one time with them and at another under its own provisions.

